# Allergen immunotherapy in asthma; what is new?

**DOI:** 10.1186/s40733-015-0006-2

**Published:** 2015-07-15

**Authors:** Giovanni Passalacqua, Anthi Rogkakou, Marcello Mincarini, Giorgio Walter Canonica

**Affiliations:** Allergy and Respiratory Diseases, IRCCS San Martino Hospital-IST-University of Genoa, Padiglione Maragliano, L.go R.Benzi 10, Genoa, 16133 Italy

**Keywords:** Allergen immunotherapy, Sublingual immunotherapy, Subcutaneous immunotherapy, Efficacy, Safety, Allergic asthma, Allergic rhinitis, Adverse events

## Abstract

The use and role of allergen immunotherapy (AIT) in asthma is still a matter of debate, and no definite recommendation about this is made in guidelines, both for the subcutaneous and sublingual routes. This is essentially due to the fact that most controlled randomised trials were not specifically designed for asthma, and that objective measures of pulmonary function were only occasionally considered. Nonetheless, in many trials, favourable results in asthma (symptoms, medication usage, bronchial reactivity) were consistently reported. There are also several meta analyses in favour of AIT, although their validity is limited by a relevant methodological heterogeneity. In addition to the crude clinical effect, a disease modifying action of AIT (prevention of asthma onset and long-lasting effects) have been reported. The safety is an important aspect to consider in asthma. Fatalities were rare: in Europe no fatality was reported in the last three decades, as in the United States in the last 4 years. Based on previous surveys, and common sense, uncontrolled asthma is still recognized as the most important risk factor for severe adverse events. On the contrary, there is no evidence that AIT can worsen or induce asthma. According to the available evidence, AIT can be safely used as add-on treatment when asthma is associated with rhinitis (a frequent condition), provided that asthma is adequately controlled by pharmacotherapy. AIT cannot be recommended or suggested as single therapy. When asthma is the unique manifestation of respiratory allergy, its use should be evaluated case by case.

## Introduction

Considering the systemic mechanisms of action of AIT [1] and the immunological unity of respiratory airways [2], it is clear that AIT is not specific for the type of disease (rhinitis or asthma) but only for the allergen causing the disease itself [3]. Nonetheless, the efficacy of AIT has been usually kept separated for asthma and rhinitis, as testified by meta-analyses, commentaries and guidelines [4–6]. Indeed, the majority of data available concern clinical trials including patients with allergic rhinitis (AR), which was the primary outcome measure, with/without asthma. Consequently, asthma-related parameters, were either secondary outcomes or subject of post-hoc analyses. This reflects the clinical practice in real life, where isolated allergic asthma without rhinitis is infrequent, but asthma is present in more than 30 % of rhinitis patients, and the majority of patients with allergic asthma have also AR [2]. Very few trials were, therefore, specifically designed to evaluate the effect of SIT in asthma alone, or with asthma parameters taken as primary outcome. This implies that few trials were adequately designed and reported, had an adequate sample size calculation and a power analysis based on asthma characteristics [7, 8]. In this regard, the best primary outcome for evaluation asthma in clinical trials is still uncertain and discussed. Asthma symptoms, asthma-free days, rescue medications usage, days free of medications, asthma-related quality of life (QoL) and asthma exacerbations are all reasonable choices [9]. Objective measures (pulmonary function test, specific and non-specific bronchial provocation, exhaled nitric oxide) would be more robust parameters, but they were considered only sporadically. Keeping in mind those methodological limitations (Table 1), the main questions are: is AIT effective in asthma?, is it safe (worsening/precipitating asthma)? is asthma a risk factor for AIT-related severe adverse events? can AIT be prescribed in patients with asthma?

**Table 1:** Main methodological limitations of the studies considering AIT in asthma

ITEM	CRITICAL ASPECTS
Patients’ selection	Patients should be selected matching asthma severity and, current asthma therapy
Primary outcome	Asthma-related parameters should be the primary outcome
Sample size calculation	Based on asthma-related primary outcome
Objective parameters	Pulmonary function tests/bronchial challenges should be included in primary outcomes
AIT protocol	Should be uniformed (duration, doses, run-in etc.)
Duration	An optimal duration of the AIT course is not established
Dose	The optimal maintenance dose needs to be established for most allergens. Discrepancies among manufacturers

The knowledge on the effects, and safety, of AIT in asthma is based, in summary, on either historical clinical trials with SCIT, previous safety surveys and recent trials with SLIT. All these data, taken together are certainly not conclusive, but some suggestions applicable in the clinical practice can be derived [7, 8].

### Ait in asthma: the clinical evidence

As mentioned above, in the large majority of clinical trials with SCIT and SLIT, rhinitis was the primary outcome studied, but in many studies part of the patients enrolled also suffered from concomitant asthma, thus, asthma-related outcomes could be analysed (for review see 3, 6–8).

A certain number of trials expecially with SLIT were, at some extent, designed to specifically investigate asthma [10–41], almost all showing significant effects on asthma symptoms, medications’ usage, or bronchial reactivity. Some studies specifically assessed the effect of AIT as inhaled corticosteroids sparing agent in asthma [21, 29, 37–40]. In the first of the two more recent studies [37], conducted with SCIT in 65 children for 8 months, a mean 50 % reduction in the dose of inhaled fluticasone was seen in the active group, who remained controlled. The other one [38] investigated 3 doses of SLIT in more than 600 adults for 1 year. With the highest dose, a significant reduction in mcg of corticosteroids was seen (p = 0.004) and the relative mean reduction was 42 %. The less recent studies involved smallest samples [21, 29, 39, 40]: overall demonstrated a steroid-sparing effect of AIT in asthma, but the extent of this effect was largely variable, and sometimes of uncertain clinical relevance. Finally, Marogna et al. [41] compared the effect, as add-on treatment, of inhaled budesonide and SLIT, and found that over a 3-year period, SLIT was overall more effective than budesonide in reducing symptoms and bronchial hyperreativity. Of note, some studies [11, 13, 21, 33, 34] reported only marginal effects on asthma, but in two of these studies all the patients (active and controls) had almost no asthma at baseline and during the trial [33, 34]. Despite the important limitations (small sample, no power calculation, variable inclusion criteria) the published studies substantially agree on the clinical efficacy of SCIT in asthma, induced by the most common allergens (grass, mite, pet dander).

Based on the results of the trials, several meta-analyses were conducted (Table 2). The largest available meta-analysis of SCIT in asthma [42] included 88 trials (70 randomized and placebo controlled, but only few with symptoms and medications clearly reported). The methodological quality was overall considered low or moderate, with only 6 trials receiving the maximum of 5 points at the Jadad score), the concealment of allocation was considered adequate in 16 trials, symptoms scores were reported in 35 studies and medications in 21. Only 20 studies included pulmonary function measurements. According to this analysis, the reduction of symptoms with mite allergens remained borderline, whereas the effect was highly significant with pollens and, in general for asthma medications. No change in pulmonary function was appreciable, but a significant improvement at the allergen-specific bronchial challenge. The heterogeneity of the studies was high, thus limiting the strength of the conclusions. Another systematic review, considering only the USA-licensed products for SCIT (15 trials with asthma symptoms reported, 790 patients) concluded there was strong evidence for efficacy of the treatment [43]. Concerning SLIT, there are two meta analyses: one including all age classes (25 randomized double blind trials, 1076 patients) [44] and one limited to pediatric subjects (9 studies, 441 patients) [45]. Both analyses confirmed the presence of a measurable effect over placebo. Nonetheless, the first metaanalysis showed no significant difference between SLIT and placebo when asthma symptoms and asthma medications were considered separately. was negative for some parameters [44]. One of the major problems of meta-analyses is that they pool together studies conducted with different allergens (i.e. mites and pollens). This aspect could be better addressed for SLIT due to the abundance of studies, restricting the analysis to mite extracts [46] or to grass extracts [47]. The meta-analysis for dust mites included 9 trials that evaluated asthma symptoms and medications. It showed a significant reduction versus placebo in symptom scores (p = 0.02) and medications (p = .02). The meta analysis for grasses did not report specific results for asthma.

**Table 2:** Meta analyses of AIT in ashtma

Author, Year (REF)	Type of AIT	Studies	PATS (A/P)^a^	Results (effect size, 95 % C.I.)
Abramson, 2010 [42]	SCIT	34 symptoms	727/557	Symptoms −0.6 (−0.83 -0.35) significant
		20 medications	485/384	Medications −0.5 (−08 -0.3) significant
Erekoshima 2013 [43]	SCIT	10 symptoms	320/308	Strength of evidence reported as “high” for both outcomes
		8 medications	285/288	
Calamita, 2007 [44]	SLIT	9 symptoms	150/153	Symptoms −0.38 (−0.79 0.03) not significant
		6 medications	132/122	Medications −0.9 (−1.9 -0.12) significant
Penagos, 2008 [45]	SLIT^ab^	9 symptoms	232/209	Symptoms −1.18 (−2.1 -018) significant
		7 medications	192/174	Medication −1.63 (−2.8 -044) significant
Compalati, 2009 [46]	SLIT mite	9 symptoms	243/209	Symptoms SMD 0.95 (1.74 0.15) significant^abc^
		7 medications	102/100	Medications SMD 1.48 (2.70 0.26) significant

^a^Active/Placebo; ^ab^only children: ^abc^Standardized mean deviation

There is still some debate about the comparison of efficacy between SLIT and SCIT. No study specifically addressed the problem for asthma, but a comprehensive view of the meta analyses suggest that the efficacy is overall the same [48]. Similarly, there is no study in asthma comparing the effects of AIT and drugs. Rak et al. [49] demonstrated the superiority of nasal corticosteroids versus AIT in rhinitis but found that AIT only could decrease the seasonal bronchial hyperreactivity in asthmatic patients and Sheikh et al. evidenced the persisting effect of SCIT over inhaled steroids after discontinuation [50]. Another trial [51] in asthmatic children showed that adding AIT to inhaled fluticasone did not cause a further improvement of symptoms, but SLIT only decreased non-bronchial symptoms. Finally, an open randomized trial of SLIT (as add-on treatment) versus inhaled budesonide alone in asthmatic patients, demonstrated an overall superiority of AIT over time [41]. All these studies that were not randomized, properly powered or were designed as open trials limits the methodological value of the observations.

### Safety aspects of ait in asthma

AIT implies the administration of extracts of substances (allergens) to which the subject is sensitized. This can lead to adverse reactions, that can be either local or systemic (SRs), this latter, spanning until anaphylaxis and death. The reported occurrence of SRs is largely variable, according to allergen, induction schedule, preparation and dose. The available data (on large populations) come from the surveys regularly performed in the USA with SCIT which [52, 53], reported about 50 deaths over a 50-year period with a risk of one death every 2.500.000 injections and one near-fatal reaction per million injections. Human errors and uncontrolled asthma were the most frequent causes of SCIT-induced adverse events [53, 54]. On the other hand, in the period 2008–2011 no further fatality due to SCIT was reported in the United States and SRs were about 0.1 % of injection visits [55]. Evaluating those data, it must be kept in mind that the practice of SCIT profoundly differs between Europe and United States, where allergen mixes and higher concentrations are commonly used [56]. Few systematic data is available in European countries [57, 58]. A recent multicenter observational study [59] suggested that systemic reactions are slightly more frequent in rhinitis with asthma than in rhinitis patients alone. According to the past observations and the few recent data, the scientific community agreed, as a prudential attitude, to consider uncontrolled/severe asthma as a major risk factor for severe adverse events of AIT [3, 5]. Despite no direct observation has been published, this attitude has been translated also to SLIT [6]. In this regard, the safety of SLIT is overall superior to that of SCIT [6, 60], at least because no fatality has been reported until now, and only few cases of suspect/ascertained anaphylaxis have been described, none directly attributable to pre-existing asthma or to worsening of asthma [61]. The potential of precipitating asthma has been considered in some studies A controlled dose-finding study of safety [62] involving 48 grass-allergic patients outside the pollen season and receiving up to 200 mcg Phl p 5, (about 40 times the SCIT dose) showed an incidence of side effects of 74 %, all of which were mild or moderate in intensity and all localized. Dahl et al. [34] assessed the safety of SLIT in asthma in more than 100 grass-allergic asthmatics. The number of side effects possibly linked to asthma (wheezing cough, dyspnoea) was similar between the active and placebo group, and there was no evidence of asthma aggravation.

According to literature, and common sense, severe/uncontrolled asthma remains the main risk factor for side effects due to AIT, although for SLIT severe asthma have been not clearly demonstrated as a specific contraindication. In general, asthma is not an absolute contraindication to AIT, if the patient is well controlled with pharmacotherapy.

As a future perspective, an uniform reporting and grading of side effects would be desirable. This has recently been addressed by the World Allergy Organization, which proposed a grading system for systemic reactions due to SCIT/SLIT [63] and for local reactions due to SLIT [64].

### Disease-modifying effects

Rhinitis is the most important independent risk factor for the development of asthma [1, 65], and in the natural history, usually rhinitis precedes asthma. AIT is an immune response modifier, thus it was hypothesized that it may alter the progression of the disease, reducing the risk of asthma onset. The preventive effect of AIT on the risk of developing asthma was quantified in some controlled trials only during the last decades. The Preventative Allergy Treatment study enrolled 205 children (aged 6–10 years) suffering from allergic rhinitis, and randomized to either drug therapy alone or drugs plus SCIT. After 3 years, the SCIT-treated patients developed significantly less asthma than the control group (odds ratio 2.5) [66]. The beneficial effect of SCIT lasted at least 7 years after discontinuation [67]. The same effect was demonstrated with SLIT. The first open controlled study [68] involved 113 children aged 5–14 years with seasonal rhinitis due to grass pollen, randomly allocated to medications plus SLIT or medications only. After 3 years, 8/45 SLIT subjects and 18/44 controls had developed asthma, with a relative risk of 3.8 for untreated patients. The other randomized open controlled trial [69] involved 216 children (5–17 years) suffering from rhinitis with/without intermittent asthma, randomly allocated 2:1 to drugs plus SLIT or drugs only. The prevalence of persistent asthma after 3 years of observation was 1.5 % in the SLIT group and 30 % for the control group.

It is true that there are only 3 studies addressing the preventive effect of AIT, involving less than 300 patients in total. Nonetheless, due to the relevance of this aspect, new trials with rigorous methodology are currently ongoing, such as the GAP study [70], which is involving more than 800 children in a 5-year double blind evaluation.

### Conclusions

The literature on the clinical and immunological effects of AIT is abundant, and many guidelines and recommendations were prepared according to structured evaluation systems such as the GRADE [71]. According to GRADE the essential requirement is that the methodology of the study is adequate (e.g. sample size, outcome, selection criteria, randomization), that is not always the case for AIT in asthma (Table 1), since many of the randomized controlled trials had important limitations (small number of patients, no objective measurement of respiratory function, no sample size calculation based on the objective parameters, variability of doses and protocols). This facts burden the meta-analyses, where the positive clinical results are counterbalanced by the high heterogeneity of the studies.

Indeed, beyond the methodological limitations, there is an abundant experimental evidence that AIT is effective in controlling symptoms and medications intake in patients with asthma (usually concomitant to rhinitis) [72]. Thus, it should be confidently stated that SLIT and SCIT can be used together with asthma medications, when asthma is associated to rhinitis and the causal role of the allergen is clearly confirmed. Also when asthma is the only allergic disease (that is rare), AIT is expected to exert a beneficial effect. It does not seem that AIT can worsen asthma, whereas uncontrolled asthma remains a significant risk factor for adverse events. The disease-modifying effects of AIT in asthma prevention should be taken into account [73], although there are so far only 3 studies on this aspect. On the other hand, the cautious attitude towards the use of AIT in asthma is testified by the fact that the recently FDA approved products (tablet SLIT for ragweed and grass) do not have the indication for asthma, but only for rhinoconjunctivitis.

Overall, it has to be considered that asthma is per se a heterogeneous disease, with different endotypes [74] and different types of underlying inflammation. In addition, allergens are often but not always the unique triggers of the inflammatory reation. These facts can explain the variable efficacy of antinflammatory treatments, allergen avoidance strategies, and AIT itself. So far, an unequivocal biomarker capable of predicting the response to treatments has not been yet identified [75].

Main unresolved questions and unclear aspects are the optimal maintenance dose to use, the duration of treatment to obtain a satisfactory long term effect, and the appropriate use of objective outcomes. Probably, the most important questions are which children with rhinitis should receive AIT to prevent the future development of asthma, and which biomarkers are relevant to identify the potential responders.
